# Prenatal identification of two discontinuous maternally inherited chromosome 7q36.3 microduplications totaling 507 kb including the sonic hedgehog gene in a fetus with multiple congenital anomalies

**DOI:** 10.1002/ccr3.982

**Published:** 2017-05-10

**Authors:** Mark Micale, Bedford Embrey, Katie Hubbell, Kelly Beaudry‐Rogers, Amy Whitten

**Affiliations:** ^1^ Department of Pathology and Laboratory MedicineBeaumont HealthRoyal OakMichigan; ^2^ Oakland University William Beaumont School of MedicineRochesterMichigan; ^3^ Department of Obstetrics and GynecologyBeaumont HealthRoyal OakMichigan

**Keywords:** Chromosome 7q duplication, chromosome microarray, incomplete penetrance, sonic hedgehog gene

## Abstract

Duplications of the *SHH* gene, an important developmental gene, are rare. Disruption of this gene produces a variable phenotype in humans from major anomalies to isolated facial defects. This is the first reported case of a maternally inherited 507 kb discontinuous chromosome 7q36.3 microduplication resulting in duplication of *SHH* and nearby enhancer sequences.

## Introduction

Duplications involving the long arm of chromosome 7 are uncommon. To date, 55 such duplications have been reported 1, with many being the unbalanced product of a balanced parental chromosome rearrangement. The phenotype of these cases is often variable because of the imbalance of another chromosome associated with the rearrangement. In contrast, twenty‐two cases of pure partial trisomy 7q have been reported 1, 2, 3, 4, 5, 6. Specifically, chromosome 7q duplications involving band 7q36.3 have been rarely reported. Most recently, a three‐generation family with agenesis of the corpus callosum and a 730‐kb duplication of 7q36.3 involving copy number gain of RNA binding motif protein 33 (*RBM33*) and sonic hedgehog (*SHH*) genes was reported 5. The phenotype of the affected family members included intellectual disability or borderline intellectual functioning, macrocephaly, a broad forehead with hypertelorism, and Chiari type I malformation. Other recent cases involve a de novo 300‐kb duplication involving the *SHH* gene in a child with muscular congenital hypertrophy 3 and a 400‐kb duplication, which included *SHH*,* CNPY1*, and *RBM33* genes in a fetus with an occipital encephalocele 6.

The *SHH* gene (OMIM 600725) localized to chromosome band 7q36.3 is an important developmental gene and is highly expressed within the developing nervous system. It has been implicated in establishment and maintenance of the left–right axis as well as being a key inductive signal in patterning of the ventral neural tube; anterior–posterior limb axis; and the developing limb bud, lungs, hindgut, and ventral somites. Disruptions of *SHH*, in general, produce a widely variable phenotype in humans from holoprosencephaly to major anomalies to isolated midline facial defects 3. Duplications of *SHH* are rare, both in affected individuals and in controls 5.

We report on a 20w6d female fetus with multiple congenital anomalies and a maternally inherited dup(7q) chromosome. One of the duplicated regions included the *SHH* gene. The fetal phenotype was consistent with duplications of this gene.

## Clinical Report and Methods

**Figure 1:**
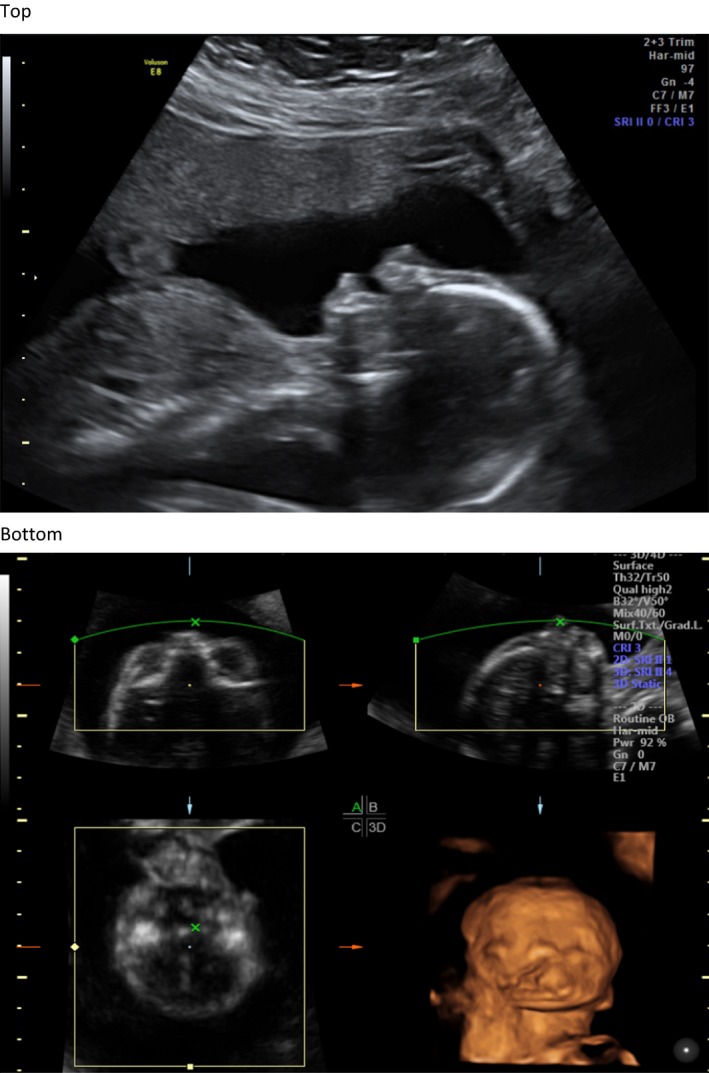
Fetal profile image showing premaxillary protrusion associated with midline cleft lip and palate (top).Three‐dimensional surface rendering in bottom right frame demonstrates large midline cleft (bottom).

**Figure 2:**
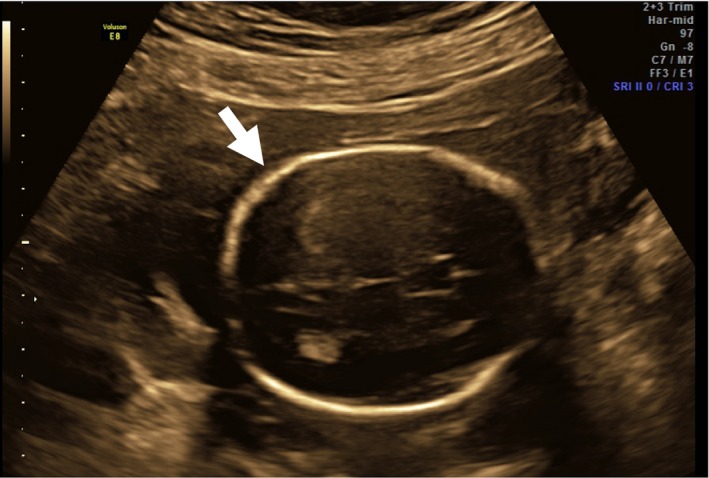
Transverse images of fetal cranium. The fetal cavum septum pellucidum is a marker for normal neurologic development, but in this image appears abnormally wide (arrow).

**Figure 3:**
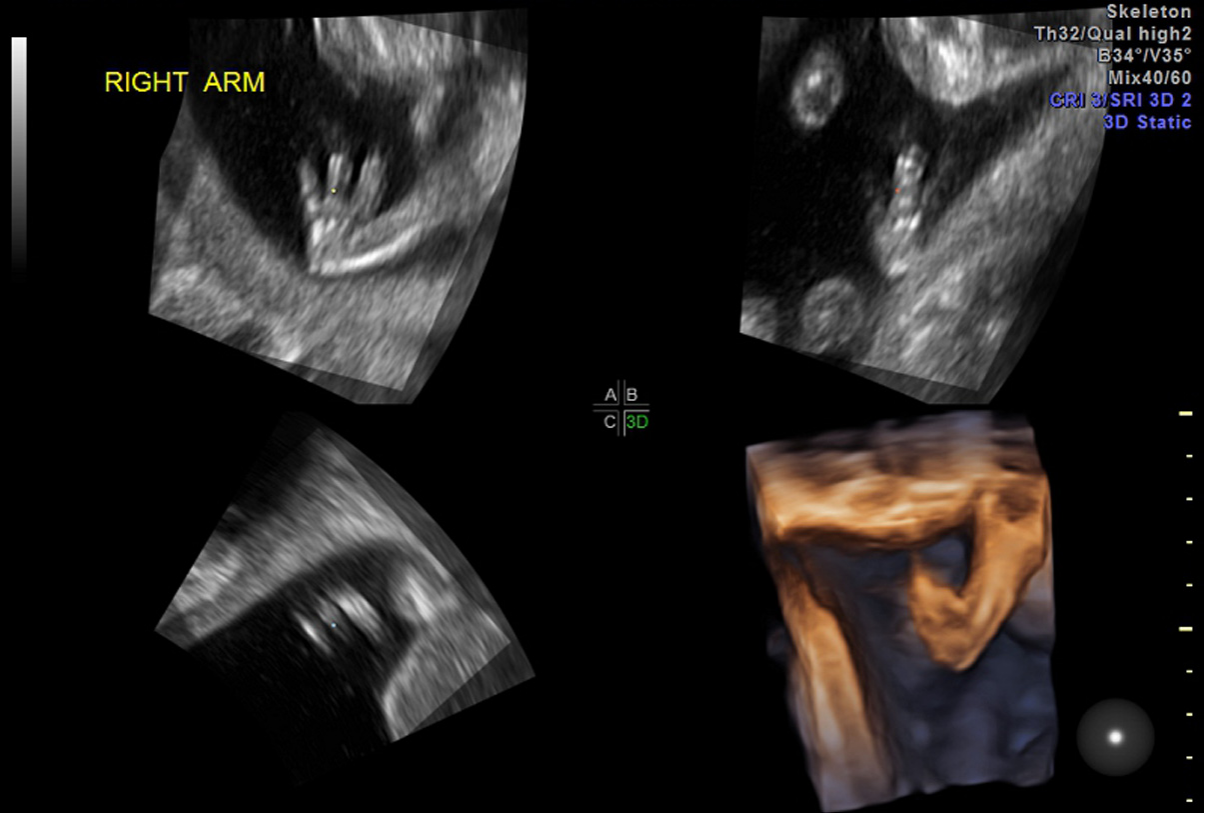
Three‐dimensional reconstruction of the right forearm. The arm is shortened with absent radius and held with abnormal joint position. The fetal thumb is absent, and fourth and fifth digits are side by side (only three distinct digits visible).

**Figure 4:**
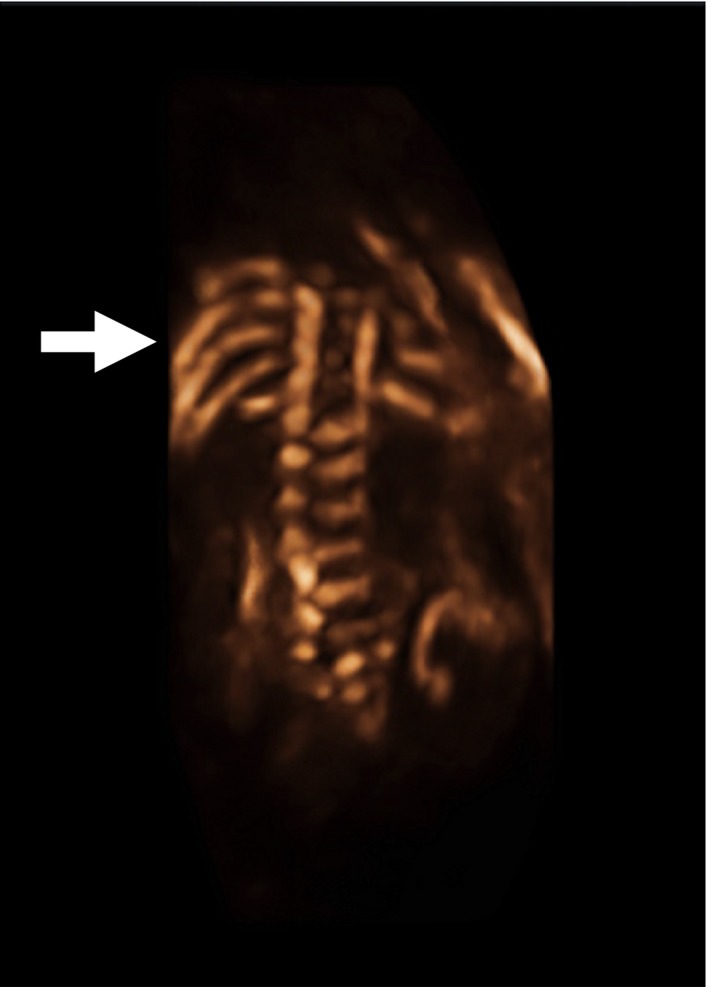
Three‐dimensional rendering of coronal view of fetal spine. There is at least one hemivertebrae and scoliosis in the lumbar and sacral spine (arrow).

The proband was the product of the first pregnancy of non‐consanguineous parents. An ultrasound revealed a large midline cleft lip/palate (Fig. 1); prominent cavum septum pellucidum without adequate visualization of the corpus callosum (Fig. 2); right‐sided heart position with normal axis; absent left kidney; absent right radius and thumb; fixed/immobile right forearm, wrist, and digits (Fig. 3); at least one hemivertebrae; and scoliosis of the sacral spine (Fig. 4).

A direct amniotic fluid SNP chromosome microarray analysis was performed utilizing the CytoScan HD SNP Array (Affymetrix, Santa Clara, CA) according to the protocol provided by the manufacturer (http://www.affymetrix.com). The array design was based on human genome build GRCH37/hg19. Microarrays were washed and stained with the Affymetrix Fluidics Station 450 and scanned with the Gene Chip Scanner 3000 using Command Console Software (Affymetrix, Santa Clara, CA). Copy number and loss of heterozygosity (LOH) analysis was performed utilizing the Chromosome Analysis Suite Version 3.0.

**Figure 5:**
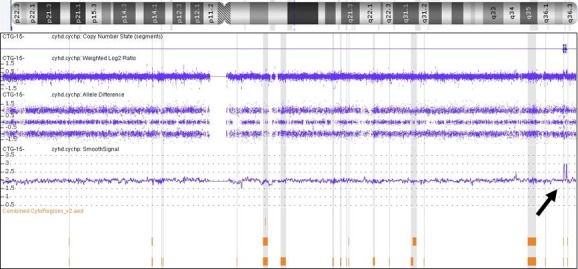
SNP chromosome microarray plot of chromosome 7 demonstrating two regions of duplication within chromosome band 7q36.3 (arrow).

**Figure 6:**
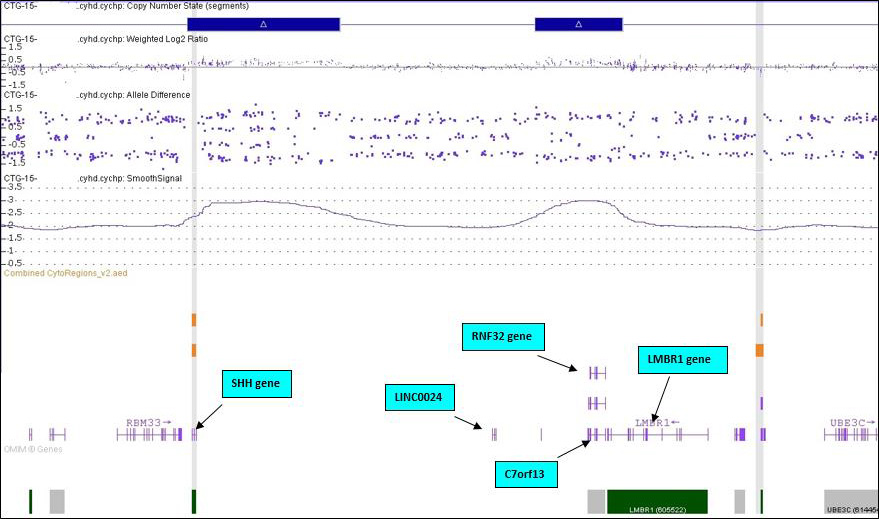
An expanded view of chromosome band 7q36.3 reveals two discontinuous duplications with location of the genes within the duplicated regions. The sonic hedgehog (*SHH*) gene lies within the more proximal duplication.

SNP array detected two discontinuous microduplications within chromosome band 7q36.3 (Figs 5 and 6). The proximal duplication measured 323 kb and contained only the sonic hedgehog (*SHH*) gene. The genomic coordinates of this duplication were arr[hg19] 7q36.3(155,586,145‐155,908,838)x3. The distal duplication measured 184 kb and contained four genes (*LINC00244, C7orf13, RNF32, LMBR1*). Its genomic coordinates were arr[hg19] 7q36.3(156,321,756‐156,505,989)x3. The genomic material separating the two duplications had good probe coverage and measured 413 kb. The couple elected to terminate the pregnancy by D&E, thus precluding an autopsy or post‐mortem MRI.

The family was initially counseled that the amniocentesis revealed two likely pathogenic duplications within chromosome 7q36.3. They were told that disruptions in the *SHH* gene have been associated with malformations in forebrain development and midline facial clefts, and as such, it was reasonable to believe that these duplications accounted for the ultrasound findings observed in the fetus. Parental SNP chromosome microarray analysis was recommended to determine if the duplications were de novo or inherited. The parents were told that, if de novo, it was more likely the duplications were causative for the abnormalities detected by ultrasound. The couple declined SNP array at the time due to insurance costs. Fetal SNP array analysis was also recommended in any future pregnancy.

In preparation for another pregnancy, the patient and spouse later had karyotype analysis (without SNP array) ordered by their primary care obstetrician. Chromosome analyses were both normal, but the couple was informed that only SNP array would be sensitive enough to detect the duplications observed in the previous pregnancy. At this time, the couple was referred back to the maternal–fetal medicine service and SNP array was performed, resulting in identification of the duplications in the phenotypically normal mother. A review of the mother's personal and family history was unremarkable for any phenotype (microform) associated with *SHH* duplication. She has two brothers without children by choice and a sister with one healthy child. Both her father and paternal grandfather have dementia, and her uncle and his son have clubfoot. The mother has since continued her care elsewhere, and it is not known whether other family members have been tested for the duplications. She understood that the duplications could have been a de novo event or could have been inherited from one of her parents. The couple was quoted a 50% risk to pass on the chromosome 7 with the duplications in any future pregnancy. It was reiterated that SNP array should be considered in any future pregnancies, that a fetus that inherits the duplications would have a wide prognostic range, including possibly a normal phenotype, and that a detailed scan and echocardiogram at 19–20 weeks of gestation would be indicated for any fetus known to have inherited the duplication.

## Discussion

The fetus described in this study presented multiple congenital anomalies including a midline defect (cleft lip/palate); dextrocardia; absent left kidney; complete right radial ray anomaly with fixed/immobile right forearm, wrist, and digits; at least one hemivertebrae; and scoliosis of the sacral spine. Clinical features described previously in pure partial trisomy 7q include developmental delay, dysmorphic facial features including cleft palate, skeletal anomalies, genitourinary tract anomalies, and heart defects including dextrocardia 2. The variable phenotype and relative lack of genotype:phenotype correlation in pure partial trisomy 7q are undoubtedly due to the rarity of this abnormality and the involvement of different affected segments of chromosome 7q. In an attempt to correlate pure partial trisomy of a specific chromosome 7q region with phenotype, a classification system has been devised which group cases into one of four categories (reviewed in Scelsa et al. 2). These include the following: group 1 – patients with duplication of the entire long arm, group 2 – heterogenous mix of large duplications spanning the long arm, group 3 – interstitial duplications of varying sizes which all have in common a proximal breakpoint between 7q21 and 7q22, and group 4 – distal duplications. The duplication identified in our patient would fit into group 4 in this scheme; however, the lack of a postmortem examination precluded a full characterization of the fetus' phenotype. In addition, the paucity of patients with a similar duplication limits identifying a common phenotype associated with 7q36 duplication.

The incomplete penetrance demonstrated in our case has been documented for many microarray‐detected genomic abnormalities and can preclude the unequivocal determination of pathogenicity of the copy number variant (CNV). Possible explanations for this genomic phenomenon include variable expressivity of the CNV in the “normal” parent, parent‐of‐origin imprinting effects, mosaicism for the CNV in the parent, a second undetectable mutation in the proband, or different allelic (more or less permissive) backgrounds in the proband and transmitting parent 7, 8. Nevertheless, the relative similarity of phenotypic features identified in other partial 7q duplications with those in our patient would support the notion that the CNV identified in the fetus was pathogenic.

The more proximal 323‐kb duplicated segment contained only the *SHH* gene. This gene codes for sonic hedgehog, one of three proteins in the hedgehog mammalian signaling pathway. This protein has inductive effects on the developing embryo. Specifically, *SHH* plays a number of key roles including ventral midline patterning of the central nervous system, establishment and maintenance of the left–right axis, and development of limb buds 9. Mutations in *SHH* are the most common cause of non‐chromosomal holoprosencephaly. Such mutations are inherited in an autosomal dominant fashion and demonstrate a widely variable phenotype, ranging from severe facial anomalies and incompatibility with extrauterine survival to mild isolated midline defects (known as microforms) such as cleft lip/palate or one maxillary central incisor 5. While the exact mechanism of this variability is currently unknown, it is believed that other gene mutations, genetic background (genetic modifiers), and environmental factors may play a role. For *SHH*, there appears to be a threshold for its signaling. Perturbations of this tightly regulated threshold, such as what can occur as a result of *SHH* deletion or duplication, or if the threshold is altered due to exposure to an environmental exposure, can result in variable disease manifestation 10. Other genes within the mammalian hedgehog signaling pathway, such as *PTCH* or *SMO*, are likely regulated as tightly and are susceptible to similar modulating factors, resulting in a phenotypic spectrum of severity.

Duplications of the *SHH* gene are rare. A familial 0.73 Mb 7q36.3 duplication involving *SHH* and *RBM33* genes was identified in four individuals from a three‐generation family with mild intellectual disability, macrocephaly, and a Chiari I malformation 5. This study also mentions four other individuals with overlapping 7q36.3 duplications found in the DECIPHER database; however, because the size of these duplications varied, no specific genotype:phenotype correlations were possible. In general, these patients presented with intellectual disability and major congenital abnormalities. A 0.30‐Mb 7q36.3 duplication involving *SHH* and *RBM33* genes was identified by oligonucleotide array in a child with congenital muscular hypertrophy 3. Lastly, a de novo 0.40‐Mb duplication of 7q36.3 involving *SHH, CNPY1*, and *RBM33* genes was identified in a patient with an occipital encephalocele 6. While the authors postulated that the duplication could have been the etiology for the patient's encephalocele given the role of sonic hedgehog in the developing brain, they conceded that there was evidence to conclude that the duplication alone might not be sufficient to cause the phenotype. The 7q36.3 duplication identified in the present case is also rare in individuals with a normal phenotype. Review of the Database of Genomic Variants (DGV) reveals only one normal individual with duplication of *SHH* and part of *RBM33* 11, and no other such duplications were found in over 15,000 control individuals 5.

Sonic hedgehog is an example of a developmental gene, which is dependent on long‐range gene regulatory mechanisms for full spatiotemporal pattern of expression. Such regulatory elements can lie hundreds of kilobases upstream or downstream of the gene itself. Specifically, *SHH* lies adjacent to a large gene desert composed of cis‐regulating enhancers, similar to other developmental genes including *SHOX, PAX6*, and *SOX9*. In the developing limb bud, *SHH* expression is restricted to a region called the zone of polarizing activity (ZPA) in the posterior limb, which sets up a morphogen gradient resulting in limb patterning. *SHH* gene expression in the ZPA is controlled by a region called ZPA‐regulatory sequence (ZRS) 12, 13. An enhancer sequence for *SHH* is located about 1 Mb upstream of the gene in intron 5 of the *LMBR1* gene, which lies within the ZRS. Point mutations within the ZRS lead to ectopic expression of *SHH* in the anterior margin of the limb bud resulting in triphalangeal thumb–polysyndactyly syndrome. While the mechanism is not entirely clear, duplication of ZRS may result in more binding sites and, thus, more bound *SHH* protein leading to aberrant expression in the limb bud 13. The duplication in our patient includes not only *LMBR1* but also the *RNF32* gene. This gene is widely expressed throughout the embryo, yet no role has yet been determined for it, although it is known that the gene contains enhancers for *SHH* located upstream and downstream, as well as residing within it 13. The distal duplication also included the *C7orf13* (chromosome 7 open‐reading frame 13) gene, an intronless gene whose protein is expressed during spermatogenesis and, thus, may play a role in sperm formation 14. Any possible role that duplication of this gene may play in our case is not obvious.

Genomic duplication of *SHH* and regulatory elements located near *SHH* such as *LMBR1* could be expected to have phenotypic consequences by altering the regulatory architecture of the genome 12. Such duplications, which can occur through either homologous or non‐homologous recombination, result in overexpression and/or ectopic expression of sonic hedgehog, and possibly other genes upstream or downstream of *SHH,* by altering the boundary of regulatory domains or by altering gene expression through novel positioning of regulatory elements. It is known that each chromosome occupies a specific area of space within the interphase nucleus known as a “chromosome territory,” which is further subdivided into compartments. Within each compartment, highly organized topologically associating domains (TAD) constrain chromatin interactions between *cis*‐regulatory elements, which occurs in a cell‐type dependent manner to modulate promoter activity by enhancers 15. The boundaries of these TADs correspond to insulator or barrier elements 12. Recent work by Symmons and her colleagues examined the effects of genomic distances within the *SHH* TAD on *SHH* expression 16. The expression of *SHH* is regulated by enhancers that span a 900‐kb genomic block, which includes ZRS. This block corresponds to an evolutionarily conserved TAD. Their study demonstrated that changing intra‐TAD distances within the *SHH* TAD, such as what could occur through duplication within chromosome region 7q36.3, had little effect on *SHH* gene expression. In contrast, disruption of the TAD, such as through an inversion, resulted in reduced long‐range interactions with a deleterious outcome. This work demonstrated that *SHH* expression was unaffected by changes in genomic distance within the TAD as long as the TAD boundaries were unaltered.

This study extends the phenotype associated with 7q36.3 duplication, which includes the *SHH* gene. The duplication identified in this study was discontinuous, and the possibility that the two duplicated segments are trans cannot be completely excluded. The pathogenicity of the duplications is not straightforward, as the phenotypically normal mother carries the same duplication. Whether the fetal phenotype is attributable primarily to the *SHH* gene duplication in this case, or is also influenced by duplication of LMBR1 and possibly RNF32 genes, is not clear.

## Authorship

MM: Laboratory medical director: wrote the manuscript. BEIV: Laboratory technologist: performed SNP array analysis. KH: Laboratory technologist: performed SNP array analysis. KBR: was the certified genetic counselor. AW: was the maternal–fetal medicine physician.

## Conflict of Interest

The authors have no conflicts of interest to disclose.
